# Surface coating affects behavior of metallic nanoparticles in a biological environment

**DOI:** 10.3762/bjnano.7.23

**Published:** 2016-02-15

**Authors:** Darija Domazet Jurašin, Marija Ćurlin, Ivona Capjak, Tea Crnković, Marija Lovrić, Michal Babič, Daniel Horák, Ivana Vinković Vrček, Srećko Gajović

**Affiliations:** 1 Division of Physical Chemistry, Ruđer Bošković Institute, Bijenička cesta 54, 10 000 Zagreb, Croatia; 2 School of Medicine, Croatian Institute for Brain Research, University of Zagreb, Šalata 3, 10 000 Zagreb, Croatia; 3 Croatian Institute of Transfusion Medicine, Petrova 3, 10 000 Zagreb, Croatia; 4 Faculty for Pharmacy and Biochemistry, University of Zagreb, Ante Kovačića 1, 10 000 Zagreb, Croatia; 5 Institute of Macromolecular Chemistry, Academy of Sciences of the Czech Republic, Heyrovský Sq. 2, 162 06 Prague 6, Czech Republic; 6 Institute for Medical Research and Occupational Health, Ksaverska cesta 2, 10 000 Zagreb, Croatia

**Keywords:** biological fluids, colloidal stability, maghemite, nanoparticles, protein interaction, silver, surface coating

## Abstract

Silver (AgNPs) and maghemite, i.e., superparamagnetic iron oxide nanoparticles (SPIONs) are promising candidates for new medical applications, which implies the need for strict information regarding their physicochemical characteristics and behavior in a biological environment. The currently developed AgNPs and SPIONs encompass a myriad of sizes and surface coatings, which affect NPs properties and may improve their biocompatibility. This study is aimed to evaluate the effects of surface coating on colloidal stability and behavior of AgNPs and SPIONs in modelled biological environments using dynamic and electrophoretic light scattering techniques, as well as transmission electron microscopy to visualize the behavior of the NP. Three dispersion media were investigated: ultrapure water (UW), biological cell culture medium without addition of protein (BM), and BM supplemented with common serum protein (BMP). The obtained results showed that different coating agents on AgNPs and SPIONs produced different stabilities in the same biological media. The combination of negative charge and high adsorption strength of coating agents proved to be important for achieving good stability of metallic NPs in electrolyte-rich fluids. Most importantly, the presence of proteins provided colloidal stabilization to metallic NPs in biological fluids regardless of their chemical composition, surface structure and surface charge. In addition, an assessment of AgNP and SPION behavior in real biological fluids, rat whole blood (WhBl) and blood plasma (BlPl), revealed that the composition of a biological medium is crucial for the colloidal stability and type of metallic NP transformation. Our results highlight the importance of physicochemical characterization and stability evaluation of metallic NPs in a variety of biological systems including as many NP properties as possible.

## Introduction

Functional nanomaterials, including nanoparticles, nanocrystals, and nanoclusters, are promising tools for new medicinal applications, particularly for clinical use in disease diagnosis and treatment [1–2]. However, only a few nanomaterials are currently in use for medical purposes [3], for example silver nanoparticles (AgNPs) and superparamagnetic iron oxide nanoparticles (SPIONs). AgNPs are exploited in medicine for biocidal therapy owing to their antibacterial, antifungal, antiviral, and anti-inflammatory properties. In addition, they attract great interest for application in a variety of other commercial products, such as mobile phones, textiles, food storage containers, refrigerators, and cosmetics [1–2]. SPIONs are exploited in numerous in vitro and in vivo biomedical applications, but the most important is their use in imaging and drug delivery systems [4]. The biomedical applications of AgNPs and SPIONs imply uptake into the body, which consequently leads to interactions with protein-containing biological fluids [5–6]. Therefore, it is of increasing interest to systematically collect detailed information on their physicochemical properties and behavior in a biological environment. Despite a considerable number of studies on the colloidal stability of AgNPs and SPIONs in cell culture media, in natural water, or in the formulation of consumer products [2,7–14], general conclusions and a clear understanding of their fate in living organisms are still lacking.

In comparison to the bulk material, the colloidal stability of the nanoparticulate form of metal is usually more complicated. In colloidal systems, there are several possible interactions between the surface atoms of NPs and the molecules present in the media like dissolution, adsorption, binding, and aggregation, all influencing biological impacts by affecting reactive oxygen species generation, cellular uptake and NP biodistribution [15–18]. Metallic NPs usually aggregate in media with high electrolyte content that correspond to biological fluids [19–27]. NP agglomeration is intended in some applications, such as in immunoassays [28], while many others require stable colloidal dispersions of NPs at high physiological ionic strength [29]. Stabilization of metallic NPs at high electrolyte content, i.e., in biological media, may be achieved by electrostatic or steric repulsions [30–32].

Various types of surface coatings have been shown to affect NP properties, particularly to improve their biocompatibility and stability against agglomeration [30,33–35]. Proteins or biologically-compatible surfactants may serve as desirable barriers preventing NPs from agglomeration in biomedical applications [18]. Moreover, when NPs enter a biological fluid, electrostatic, dispersive, and covalent interactions cause proteins to adsorb on NP surfaces, leading to the formation of a dynamic protein corona [30,36–38]. The nature and the concentration of these proteins not only determine the behavior and biological identity of the NPs, but consequently biouptake, biodistribution and possible unwanted biological side effects [39–41]. It has already been shown that the size of the NP correlates with the uptake and toxicity of metallic NPs [41–42], whereas differences in surface coatings influence cytotoxicity and surface charge [43]. However, it is still unclear how different surface coatings affect the interaction of NPs with biological environments and the formation of the protein corona.

Because AgNPs and SPIONs with various coatings are used in many nanotherapeutic and consumer products [44], it has become critical to fill the knowledge gap surrounding the mechanisms of colloidal destabilization including the role of surface coating in the biocompatibility of metallic NP. The systematically collected and thoroughly analyzed data presented in this study will provide further insight into the behavior of AgNPs and SPIONs in complex biological media and the influence of surface properties on their colloidal stability. Furthermore, the obtained results contribute to the understanding of principal factors governing the behavior of metallic NPs in modelled and real biological fluids.

**Figure 1:**
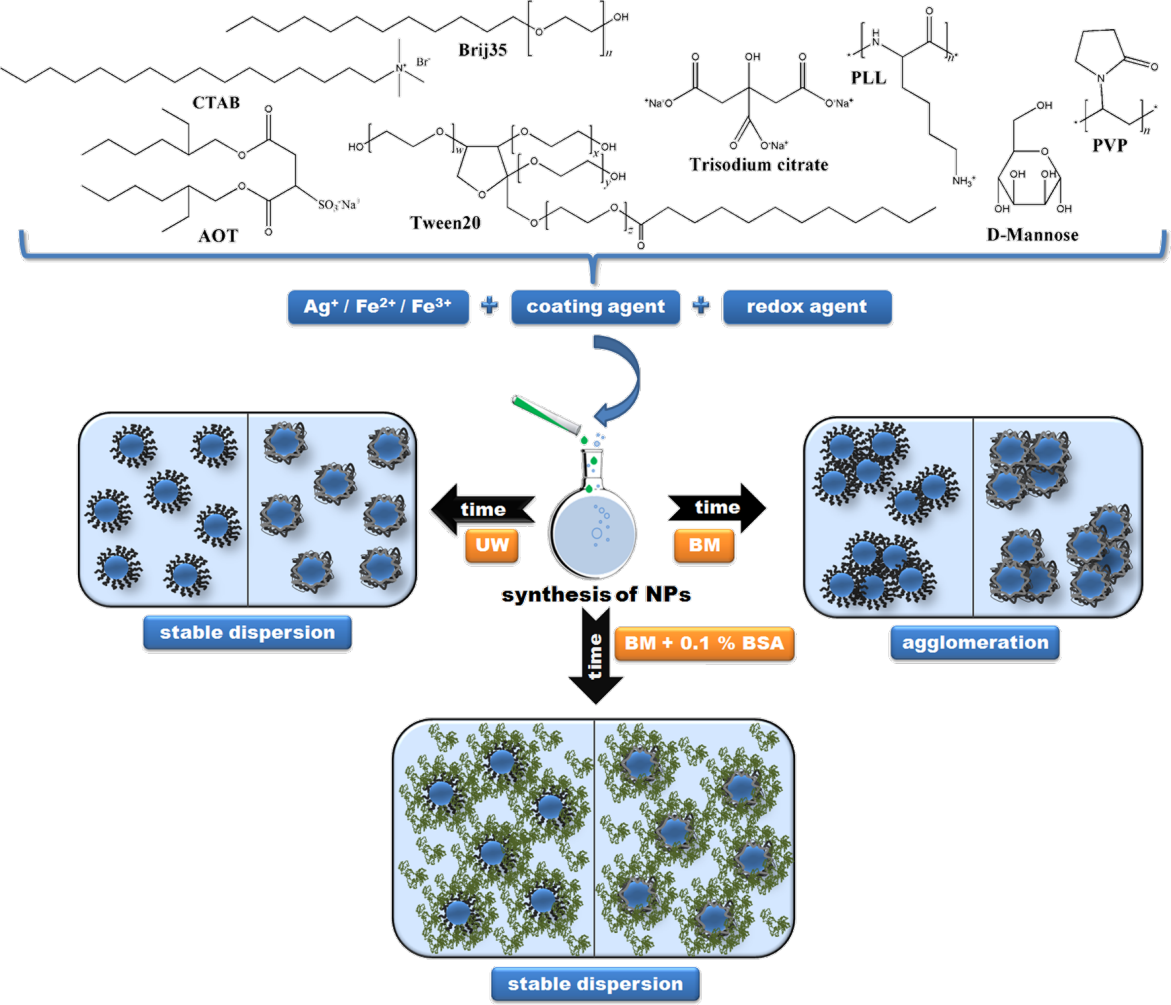
Experimental setup for stability evaluation of differently coated metallic nanoparticles in different media (UW - ultrapure water, BM - biological cell culture medium without addition of protein, BMP - BM supplemented with common serum protein).

The aim of this study was to analyze the colloidal stability and behavior of differently coated AgNPs and SPIONs under conditions close to those found in biological fluids. A systematic investigation was performed using a set of eight kinds of AgNPs and three kinds of SPIONs, each of similar size but stabilized with different surface coatings. For the purpose of systematic investigation, surface coatings were chosen following several criteria: (a) to include non-ionic as well as positively and negatively charged coatings, (b) to employ coatings of different chemical functionality, i.e., polymers, surfactants, small ionic molecules, (c) to include coatings of different hydrophilic–hydrophobic balance in molecular structure. The selected coating agents enabled us to investigate the influence of electrostatic and/or steric effects on the stabilization of NPs. Thus, AgNPs were produced with the following coatings (Figure 1): trisodium citrate (CITAgNP), sodium bis(2-ethylhexyl) sulfosuccinate (AOTAgNP), cetyltrimethylammonium bromide (CTAAgNP), poly(vinylpyrrolidone) (PVPAgNP), poly(L-lysine) (PLLAgNP), bovine serum albumin (BSAAgNPs), Brij 35 **(**BrijAgNP) and Tween 20 (TweenAgNP). The SPIONs were prepared as uncoated γ-Fe_2_O_3_ NPs (UNSPIONs), and coated with D-mannose (MANSPIONs) or poly(L-lysine) (PLLSPIONs). Three media for NP dispersion were investigated: ultrapure water (UW), biological cell culture medium without addition of protein (BM), and BM supplemented with common serum protein (BMP). In addition, the behavior of NPs was investigated in real biological fluids: whole blood (WhBl) and blood plasma (BlPl) taken from Wistar rats. Dulbecco’s modified Eagle’s medium (DMEM), as a common cell culture medium for a broad range of mammalian cells, was used as a model biological fluid. Bovine serum albumin (BSA) served as a model serum protein due to its biological relevance and high significance in biomedical applications. Albumin is the most abundant protein in mammalian blood plasma and has outstanding buffering ability [3,45]. In addressing the effects of surface coating on the stability and behavior of NPs in selected model biological environments, three types of widely used measurement techniques were employed: dynamic light scattering (DLS), electrophoretic light scattering (ELS) and transmission electron microscopy (TEM). We expect our results to be applicable in a wide variety of different NP types, allowing for robust interpretation and predictive tools in nanobioscience and nanobiotechnology.

## Results and Discussion

In this study, the role of surface coating agents on the behavior of well-characterized silver and superparamagnetic iron oxide NPs [46] in different biological environments was investigated in adherence to the experimental scheme presented in Figure 1.

### Characteristics of prepared AgNPs and SPIONs

**Figure 2:**
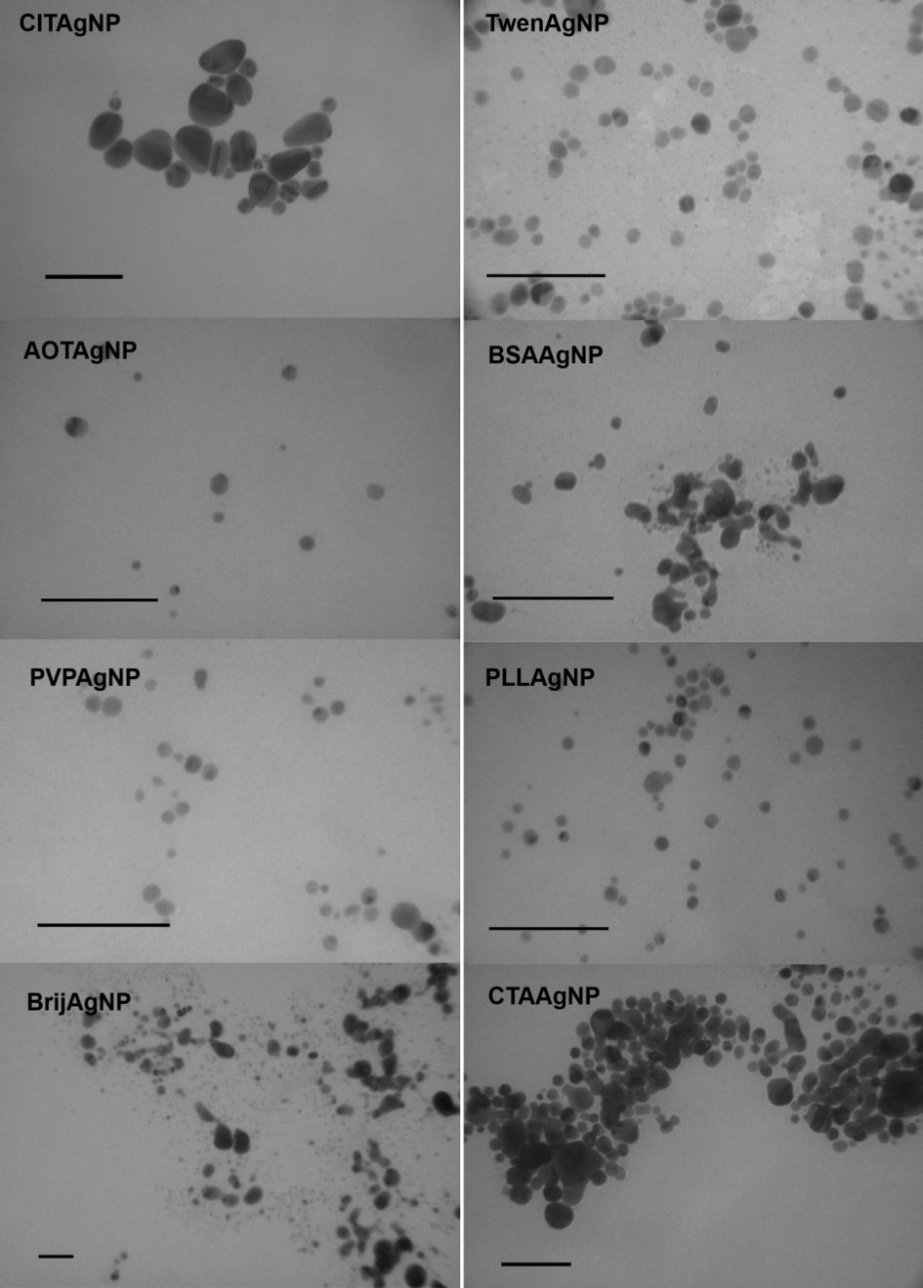
Transmission electron micrographs (TEM) of different silver nanoparticles coated with trisodium citrate (CITAgNP), sodium bis(2-ethylhexyl) sulfosuccinate (AOTAgNP), poly(vinylpyrrolidone) (PVPAgNP), Brij 35 (BrijAgNP), Tween 20 (TweenAgNP), bovine serum albumin (BSAAgNP), poly(L-lysine) (PLLAgNP), and cetyltrimethylammonium bromide (CTAAgNP). Scale bars are 100 nm.

As the first step, the physicochemical properties of freshly synthesized NPs were carefully evaluated in UW using DLS, ELS and TEM. Table 1 and Table 2 summarize the hydrodynamic diameters (*d*_H_) obtained for both volume- and intensity-weighted distributions of differently coated AgNPs and SPIONs under study. DLS measurements in UW showed that both the volume and the intensity size distributions were monomodal only for AOTAgNPs. Volume-weighted size distributions were bimodal for all of the other investigated NPs, with the exception of CTAAgNPs for which distributions showed three peak maximums. In terms of size, the majority of AgNPs had a *d*_H_ that ranged from 5 to 25 nm. As expected, the intensity-based size distributions yielded different results than the volume-based values. For example, a volume of more than 95% of PLLAgNPs had a mean size of 7.4 ± 1.3 nm, while intensity-weighted distribution showed a mean value of 115.7 ± 15.4 for ca. 88.1% of PLLAgNPs (Table 1). As already noted, it is known that intensity size distributions are subject to scattering interferences because the intensity of scatter light is much greater for large agglomerates compared to small particles. In accordance with the obtained DLS data, TEM images in UW showed monodisperse AOTAgNPs, PVPAgNPs, TweenAgNPs and PLLAgNPs, whereas other AgNPs were polydispersed (Figure 2). All of the AgNPs visualized by the TEM were spherically shaped except rod-like CITAgNPs (Figure 2).

**Table 1:** Hydrodynamic diameter (*d*_H_) obtained from size distributions by volume and intensity of differently coated silver nanoparticles in ultrapure water (UW) and biological medium (BM) after 1 h at 25 °C. Coating agents: trisodium citrate (CITAgNP), sodium bis(2-ethylhexyl) sulfosuccinate (AOTAgNP), poly(vinylpyrrolidone) (PVPAgNP), Brij 35 (BrijAgNP), Tween 20 (TweenAgNP), bovine serum albumin (BSAAgNP), poly(L-lysine) (PLLAgNP), and cetyltrimethylammonium bromide (CTAAgNP).

NPs type	medium	*d*_H_ (nm)	% mean volume	*d*_H_ (nm)	% mean intensity

CITAgNPs	UW	12.1 ± 2.8	97.1	15.0 ± 1.8	11.6
		96.3 ± 10.3	2.7	144.3 ± 11.7	87.8

	BM	13.4 ± 2.5	85.5	16.1 ± 2.9	8.0
		63.3 ± 7.2	14.1	101.4 ± 16.3	89.9

AOTAgNPs	UW	19.9 ± 0.5	99.4	27.8 ± 0.4	96.5

	BM	409.0 ± 74.1	93.2	295.4 ± 75.0	98.4
		5351 ± 128	7.3	5144 ± 228	2.0

PVPAgNPs	UW	4.9 ± 1.7	98.7	6.2 ± 1.1	11.3
		33.5 ± 4.0	1.2	69.5 ± 2.9	86.5

	BM	4.1 ± 1.3	98.5	5.4 ± 1.4	9.2
		37.9 ± 2.6	1.6	78.9 ± 10.7	89.4

BrijAgNPs	UW	24.1 ± 14.3	62.1	26.9 ± 2.1	2.4
		129.3 ± 56.4	37.6	164.7 ± 4.8	97.4

	BM	269.5 ± 25.6	82.5	246.9 ± 19.2	91.5
		56.5 ± 7.9	10.2	56.5 ± 10.8	3.4
		5220 ± 126	7.5	4974 ± 219	4.5

TweenAgNPs	UW	5.5 ± 0.3	98.8	6.9 ± 0.5	20.4
		36.1 ± 2.5	1.2	68.9 ± 3.6	79.3

	BM	11.3 ± 2.4	92.6	13.7 ± 3.1	7.8
		98.3 ± 15.9	4.5	143.7 ± 38.9	84.2
		5019 ± 307	3.7	4448 ± 543	7.2

BSAAgNPs	UW	12.8 ± 8.1	89.8	85.9 ± 22.3	96.7
		65.7 ± 26.1	8.7	12.4 ± 0.8	1.8

	BM	15.6 ± 4.4	60.6	25.1 ± 14.5	13.4
		47.3 ± 8.7	84.8	128.6 ± 28.8	86.5

PLLAgNPs	UW	7.4 ± 1.3	96.2	8.9 ± 1.7	2.9
		55.7 ± 13.4	3.7	115.7 ± 15.4	88.1

	BM	686.6 ± 133.8	95.0	542.4 ± 135.7	97.1
		5289 ± 214	4.7	5038 ± 105	2.8

CTAAgNPs	UW	17.4 ± 5.4	88.1	22.3 ± 5.7	6.0
		81.5 ± 7.6	2.9	—	—
		193.6 ± 36.8	8.7	182.9 ± 17.4	91.6

	BM	27.9 ± 5.4	40.9	32.3 ± 6.7	4.2
		71.8 ± 7.1	10.6	—	—
		602.0 ± 57.2	51.4	418.8 ± 72.7	95.6

**Table 2:** Hydrodynamic diameter (*d*_H_) obtained from size distributions by volume and intensity of uncoated superparamagnetic iron oxide nanoparticles (UNSPIONs) and coated with poly(L-lysine) (PLLSPIONNs) or D-mannose (MANSPION) in ultrapure water (UW) and biological medium (BM) after 1 h at 25 °C.

NPs type	medium	*d*_H_ (nm)	% mean volume	*d*_H_ (nm)	% mean intensity

UNSPIONs	UW	62.0 ± 13.8	77.1	48.9 ± 19.4	82.3
		105.6 ± 28.8	22.3	130.3 ± 30.0	17.1

	BM	765.7 ± 170.4	95.1	670.7 ± 200.1	92.5
		5380.5 ± 72.3	4.6	5060.2 ± 899.9	6.8

PLLSPIONs	UW	50.7 ± 24.2	37.7	53.6 ± 23.4	44.8
		279.7 ± 144.2	61.6	254.8 ± 112.4	54.9

	BM	138.8 ± 35.1	16.3	153.3 ± 42.2	35.7
		688.6 ± 167.3	76.7	610.4 ± 185.6	61.3

MANSPIONs	UW	43.8 ± 20.9	85.8	70.9 ± 39.3	97.6
		130.3 ± 30.1	15.9	—	—

	BM	131.6 ± 29.7	1.6	137.6 ± 29.3	5.3
		723.0 ± 170.6	93.7	636.2 ± 193.6	93.7
		5322.4 ± 198.3	4.8	5209.7 ± 192.1	1.0

**Figure 3:**
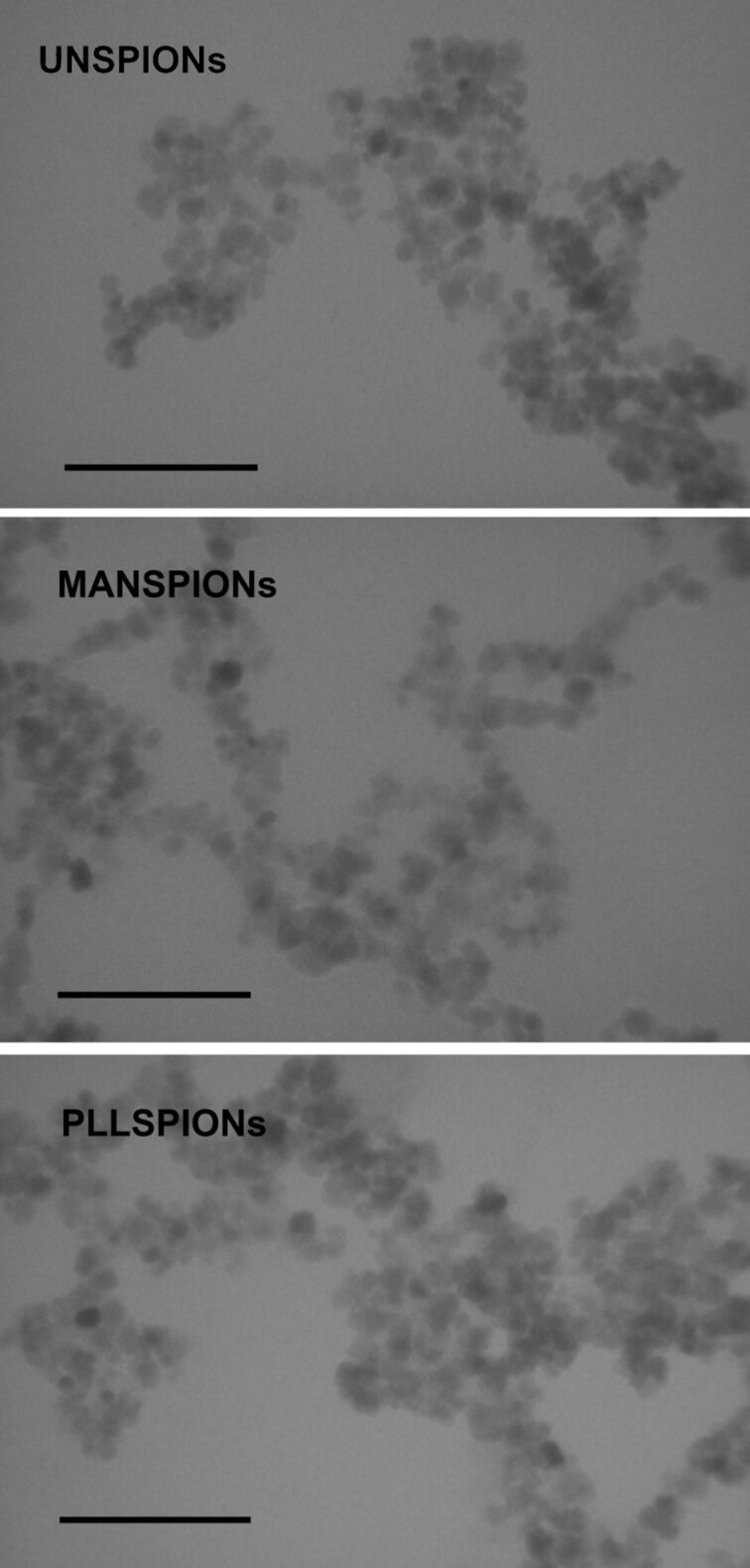
Transmission electron micrographs (TEM) of differently coated superparamagnetic iron oxide nanoparticles: uncoated (UNSPION) and coated with D-mannose (MANSPION) and poly(L-lysine) (PLLSPION). Scale bars are 100 nm.

The DLS measurements showed that SPIONs had two particle populations in UW: one with *d*_H_ of about 50 nm and another with *d*_H_ larger than 100 nm at peak maximum (Table 2). The initially synthesized SPIONs were much smaller than 10 nm as observed by TEM [4,47]. The results presented in Table 2 and Figure 3 clearly indicate a formation of SPION aggregates in UW. As it has been documented that SPIONs lose colloidal stability over time [47], the aggregates were expected considering that the SPIONs were synthesized 40–60 days prior to DLS and TEM experiments.

**Figure 4:**
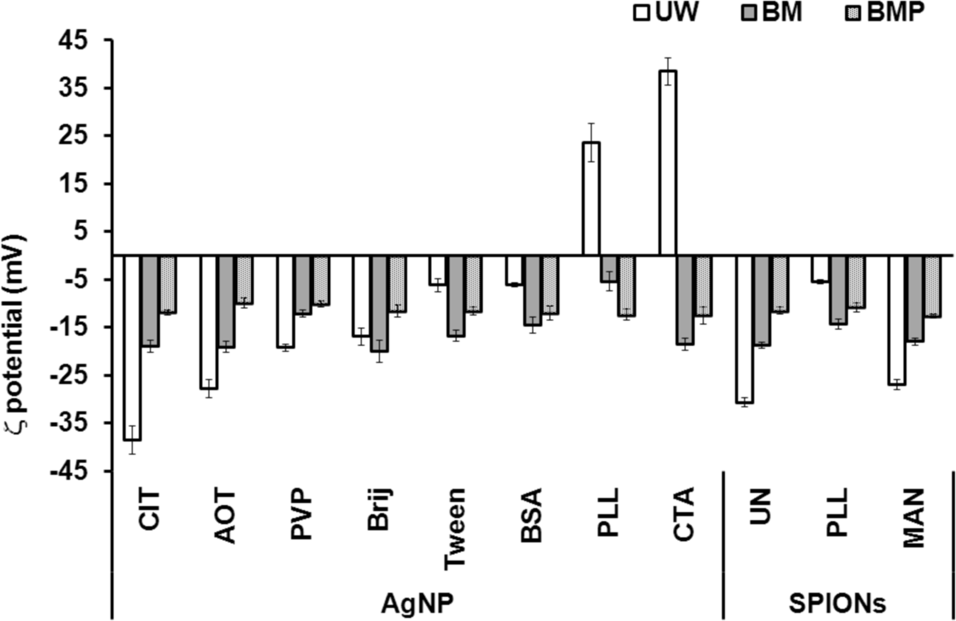
Zeta-potential (ζ) values of differently coated silver (AgNPs) and superparamagnetic iron oxide nanoparticles (SPIONs) in ultrapure water (UW), biological medium (BM) and biological medium supplemented with 0.1% bovine serum albumin (BMP) after 1 h at 25 °C. Coating agents: trisodium citrate (CIT), sodium bis(2-ethylhexyl) sulfosuccinate (AOT), poly(vinylpyrrolidone) (PVP), Brij 35 (Brij), Tween 20 (Tween), bovine serum albumin (BSA), poly(L-lysine) (PLL), cetyltrimethylammonium bromide (CTA) and D-mannose (MAN).

The ELS data, presented in Figure 4, showed that nine out of the eleven studied NPs had negative ζ-potential in UW, although one of our “synthetic” goals was to prepare positively, neutral and negatively coated, i.e., charged metallic NPs (Figure 1). The coating of AgNPs with PLL and CTA led to positively charged AgNPs characterized by ζ-potentials of 23.6 ± 4.0 and 38.5 ± 2.9 mV, respectively. Both of these coating agents are positively charged at pH 6–7, which was used in this study. For PLLSPIONs, the negatively charged surface of the maghemite core was only partially compensated by positive PLL resulting in a ζ-potential value of −5.4 ± 0.4 mV. As expected, the use of negatively charged coating agents CIT, AOT and MAN resulted in NPs bearing an overall negative charge of −38.5 ± 2.9, −27.8 ± 1.9 and −26.9 ± 1.1 mV, respectively. For AgNPs coated with uncharged molecules; PVP, Tween 20 and Brij 35, we expected a ζ-potential close to zero. However, all of the three mentioned AgNPs were characterized by slightly negative ζ-potentials ranging from −6 to −19 mV. Since we employed a borohydride reduction of AgNO_3_ during AgNP synthesis, the 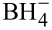 anions left over after synthesis were obviously attached next to the surface coatings of PVPAgNPs, BrijAgNPs and TweenAgNPs and led to a slightly negative ζ-potential. Synthesis of AgNPs using BSA as coating agent also resulted in NPs with a slightly negative surface charge.

### Agglomeration behavior of different AgNPs and SPIONs in biological media

The DLS and ELS methods were used to quantify the agglomeration of differently coated AgNPs and SPIONs in DMEM, a model biological media (BM), while TEM provided a visual presentation of NPs. Although the terms aggregation and agglomeration are used interchangeably, this study uses the term agglomeration because many recent studies have shown that NPs tend to agglomerate in aqueous biological matrices characterized by high ionic strength and neutral pH, such as phosphate-buffered saline and cell culture media [19–26,30]. The term aggregation indicates strongly bonded or fused particles and agglomeration indicates more weakly bonded particles.

**Figure 5:**
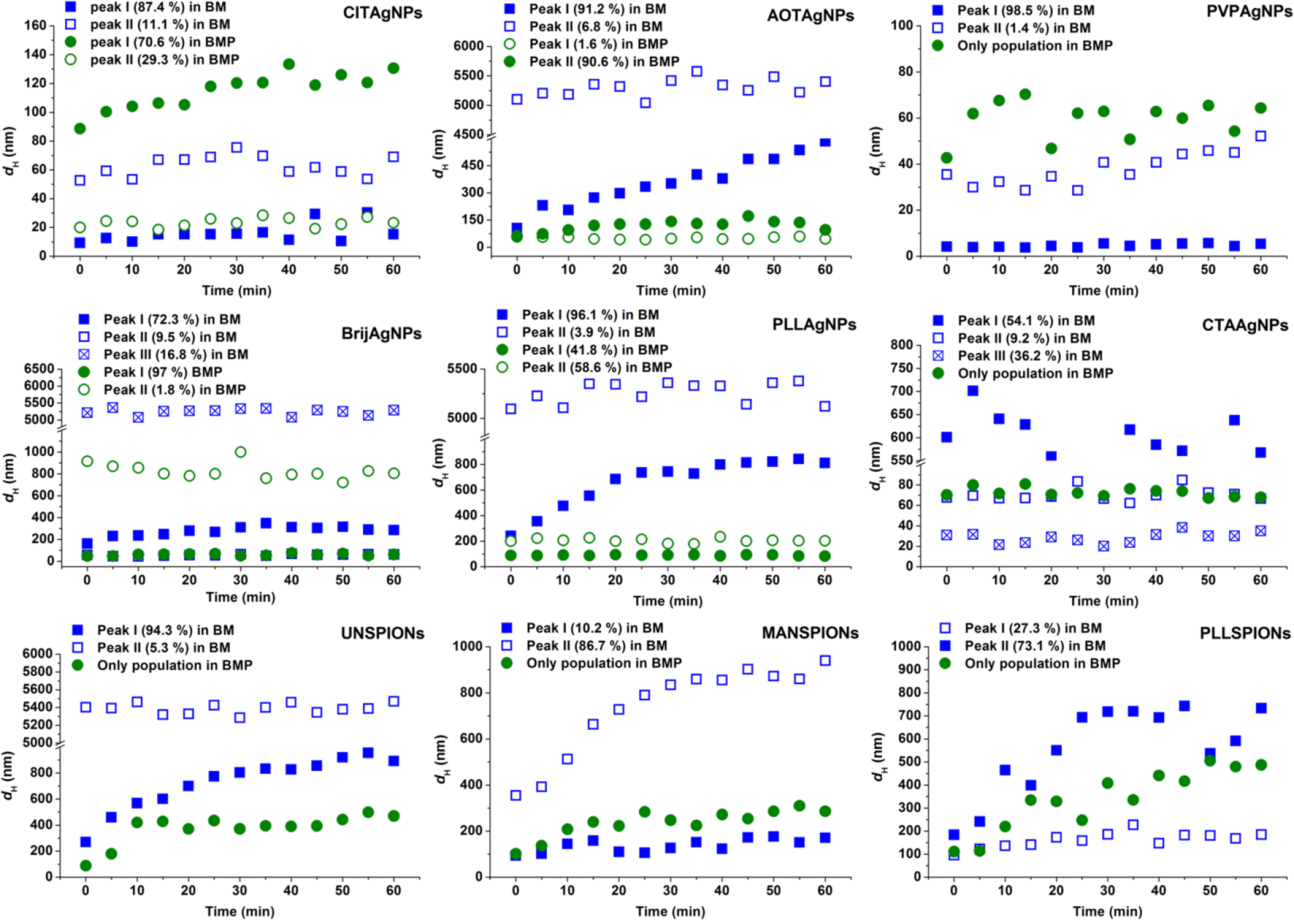
Temporal evolution of the hydrodynamic diameter (*d*_H_) obtained from size distributions by volume of differently coated metallic nanoparticles in biological media (BM) and biological media supplemented with 0.1% bovine serum albumin (BMP) over a period of 1 h at 25 °C. Results are presented for silver nanoparticles coated with trisodium citrate (CITAgNP), sodium bis(2-ethylhexyl) sulfosuccinate (AOTAgNP), poly(vinylpyrrolidone) (PVPAgNP), Brij 35 (BrijAgNP), poly(L-lysine) (PLLAgNP)**,** cetyltrimethylammonium bromide (CTAAgNP); and superparamagnetic iron oxide nanoparticles uncoated (UNSPION) and coated with D-mannose (MANSPIONs) and poly(L-lysine) (PLLSPION).

**Figure 6:**
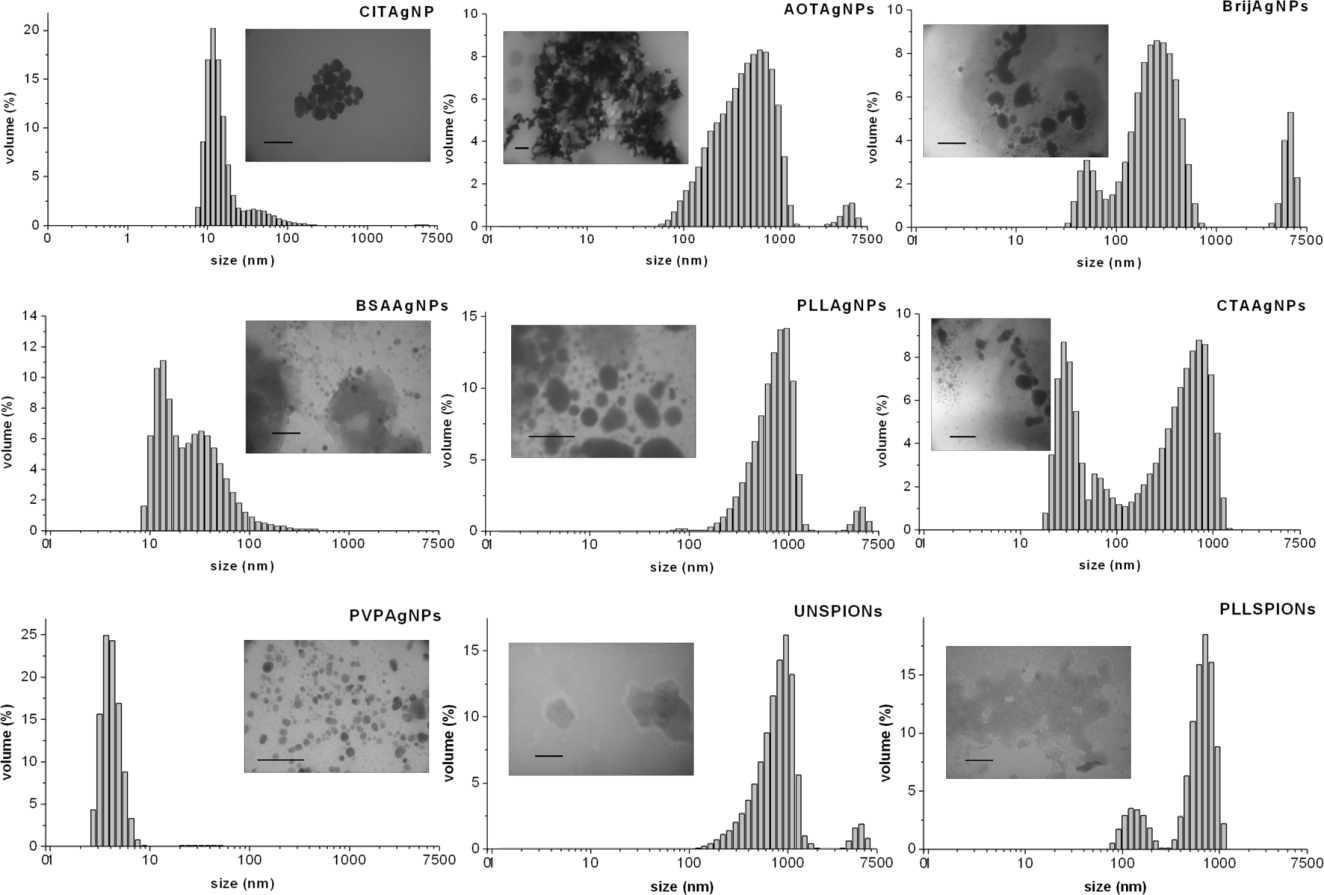
Transmission electron micrographs (TEM) and corresponding size distributions by volume of differently coated metallic nanoparticles in biological media after 1 h at 25 °C. Results are presented for silver nanoparticles coated with trisodium citrate (CITAgNP), sodium bis(2-ethylhexyl) sulfosuccinate (AOTAgNP), Brij 35 (BrijAgNPs), bovine serum albumin (BSAAgNPs), poly(L-lysine) (PLLAgNP)**,** cetyltrimethylammonium bromide (CTAAgNP), and poly(vinylpyrrolidone) (PVPAgNP) and superparamagnetic iron oxide nanoparticles uncoated (UNSPION) and coated with poly(L-lysine) (PLLSPION). Scale bars are 100 nm.

All of the obtained results for the agglomeration of differently coated AgNPs and SPIONs in BM are given in Table 1 and Figures 4–6. One would have expected that the stabilization would have been more effective using ionic coating agents when compared to non-ionic surfactants and polymers, but the explanation for the agglomeration behavior of the investigated AgNPs and SPIONs is not as straightforward. For CITAgNPs, PVPAgNPs and BSAAgNPs, the results clearly showed good colloidal stability, i.e., the size distributions in BM were similar to those obtained for dispersions in UW (Table 1).

However, the absolute value of the ζ-potential for BSAAgNPs increased after dispersion in BM. Conversely, the ζ-potential for CITAgNPs and PVPAgNPs decreased compared to that measured in UW (Figure 4). It would be expected that, when the ζ-potential approaches zero, interparticle repulsion decreases as does the stability of the dispersion. CITAgNPs are stabilized primarily through electrostatic repulsions, while bulky ligands such as PVP and BSA provide additional steric hindrances. Our results, in accordance with previously published data [48], imply that CIT, PVP and BSA provided colloidal stability for AgNPs irrespective to the type of surface interactions. It is interesting that we have recently observed [49] a significant agglomeration of CITAgNPs in phenol red-free DMEM (product number 12-709, Lonza, Verviers, Belgium) and in RPMI-1640 medium (product number R5886, Sigma-Aldrich, Munich, Germany) [50]. Both of these formulations contained HEPES as the buffering agent, while the media used in the recent work, where much like in this study no agglomeration of CITAgNPs [48] was observed, were buffered with phosphate buffer (PB). The most common buffering agents are PB and HEPES, which significantly differ in their chemical composition. Consequently, the behavior and stability of NPs in PB might be completely different from that in HEPES buffering system [21]. As phosphate, and not HEPES, is a normal constituent of mammalian blood and other body fluids, DMEM buffered with PB was chosen as the model BM.

Other AgNPs and all of the SPIONs agglomerated almost immediately after addition to the media, as can be seen from Figure 5. Thus, the high ionic strength of BM caused an agglomeration expected to be close to diffusion-limited. Moreover, the fast agglomeration of investigated NPs in BM was visible even to the naked eye. Immediately after the addition of clear NP stock solution into the BM, a cloudy black precipitate was observed at the bottom of the flask. AOTAgNPs, BrijAgNPs, PLLAgNPs, UNSPIONs and MANSPIONs showed the most pronounced agglomeration in BM and clusters of ca. 5 µm were observed (Table 1, Figure 6). As can be seen from the TEM micrographs and corresponding size distributions (Figure 6), agglomerated NPs are characterized by very high polydispersity. The CTAB and Tween coatings prevented severe agglomeration of AgNPs in BM due to steric repulsion effects. According to the volume-weighted size distribution data, less than 10% of TweenAgNPs population agglomerated in BM, while 50% of CTAAgNPs was agglomerated to clusters of about 600 nm (Table 1). Interestingly, PLL prevented a harsh agglomeration of SPIONs, but not of the AgNPs. It is known that NP coating agents can lose their stabilizing effect at high ionic strength due to complexation with counter ions. Consequently, van der Waals attraction forces induce aggregation of unprotected NPs [3]. Thus, the chemical nature of the surface-capping agents played a significant role in the conservation of colloidal stability of metallic NPs in BM. The observed differences in ζ-potential in BM compared to UW provide an additional important explanation [21]. Dispersion in BM resulted in a net-negatively-charged layer on the surfaces of all of the studied NPs, while absolute values of the ζ-potential were decreased in BM for all NPs except for BrijAgNPs, TweenAgNPs, BSAAgNPs and PLLSPIONs. This observation may explain the good colloidal stability of BSAAgNPs and PLLSPIONs in BM, moderate stability of TweenAgNPs, and instability of PLLAgNPs, but is contradictory to the observed behavior of BrijAgNPs. The electrostatic stabilization effect, playing the key role for CITAgNPs was also important for PVPAgNPs characterized by the negative ζ-potential imparted by adsorbed 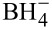, a residual side product from AgNPs synthesis. In addition, PVPAgNPs were stabilized by steric repulsion of PVP molecules. However, 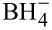 anions were also attached to the BrijAgNPs and TweenAgNPs coatings, but adsorption of Tween 20 and Brij 35 surfactants to the AgNPs surfaces was much weaker, thus providing lower colloidal stability compared to PVP. This has already been described previously [51]. It is important to note that charge reversal was noticed in the ζ-potential measurements of positively charged PLLAgNPs and CTAAgNP after dispersion in BM, which affected their stability and heavily increased the possibility of their agglomeration. Lower stability of PLLAgNPs compared to CTAAgNPs could be explained by the much more negative ζ-potential value of CTAAgNPs in BM. The DLS results agree well with the complementary information obtained by TEM observation (Figure 6). The TEM images provide evidence that no changes in the morphology or size of the CITAgNPs, PVPAgNPs and BSAAgNPs occurred upon dispersion in BM. Conversely, after being dispersed in the BM, all of the other studied NPs exhibited disordered and agglomerated morphologies (Figure 6). Small AgNP nanospheres coated with AOT, Brij, Tween, PLL and CTAB, and SPIONs were strongly damaged and had irregular surfaces (Figure 6).

In summary, different coating agents used on AgNPs and SPIONs imparted different colloidal stabilities in the same biological media. The obtained data clearly show that a combination of negative charge and high adsorption strength of coating agents alongside molecular structure are important factors that impart good colloidal stability of metallic NPs in electrolyte-rich fluids. Moreover, DLS, ELS and TEM proved to be sufficient and fast screening methods for a colloidal stability evaluation of metallic NPs in biological environments.

### Effect of albumin on the dispersibility of AgNPs and SPIONs in biological media

When suspended in biological fluids, NPs rapidly interact with proteins that form a dynamical layer all over the NP surface, known as a protein corona (PC) [36–37,39]. Subsequently, the formation of PC modifies the physicochemical properties of NPs, while proteins may undergo conformational and functional changes [52–56]. Consequently, the presence of proteins in dispersion media alters the physicochemical behavior and stability of NPs. DLS data, shown in Table 3 and Figure 5, suggest that BSA stabilized the dispersion of both types of studied metallic NPs in BMP.

**Table 3:** Hydrodynamic diameter (*d*_H_) obtained from size distributions by volume and intensity of differently coated silver (AgNPs) and superparamagnetic iron oxide nanoparticles (SPIONs) in biological medium supplemented with 0.1% bovine serum albumin (BMP) after 1 h at 25 °C. Coating agents: trisodium citrate (CIT), sodium bis(2-ethylhexyl) sulfosuccinate (AOT), poly(vinylpyrrolidone) (PVP), Brij 35 (Brij), Tween 20 (Tween), bovine serum albumin (BSA), poly(L-lysine) (PLL), cetyltrimethylammonium bromide (CTA) and D-mannose (MAN).

NPs type	*d*_H_ (nm)	% mean vol	*d*_H_ (nm)	% mean intensity

CITAgNPs	114.2 ± 12.9	82.3	117.8 ± 9.5	99.9
	22.4 ± 2.9	17.7	—	—

AOTAgNPs	47.8 ± 8.9	1.7	63.4 ± 27.0	7.1
	671.0 ± 140.4	0.1	548.9 ± 126.4	20.4

PVPAgNPs	59.6 ± 11.4	0.2	82.9 ± 20.7	36.2

BrijAgNPs	59.2 ± 9.4	2.7	91.7 ± 14.0	74.6
	848.3 ± 9.6	0.3	—	—
	4304 ± 204	0.3	4882 ± 187	8.9

TweenAgNPs	55.2 ± 6.8	0.9	87.8 ± 10.9	70.4

BSAAgNPs	86.5 ± 17.5	0.2	124.6 ± 26.7	41.4

PLLAgNPs	85.6 ± 17.6	41.2	34.3 ± 7.8	2.3
	208.4 ± 14.8	56.7	174.9 ± 8.4	97.7

CTAAgNPs	71.8 ± 6.4	0.9	99.7 ± 10.6	72.5

UNSPIONs	43.1 ± 6.4	0.1	54.7 ± 19.4	4.3
	417.6 ± 41.7	0.1	489.8 ± 69.7	29.7

PLLSPIONs	537.9 ± 64.3	0.3	382.1 ± 28.5	69.8

MANSPIONs	30.5 ± 15.9	0.1	31.8 ± 14.8	3.1
	778.1 ± 179.7	0.3	680.4 ± 237.7	41.3

**Figure 7:**
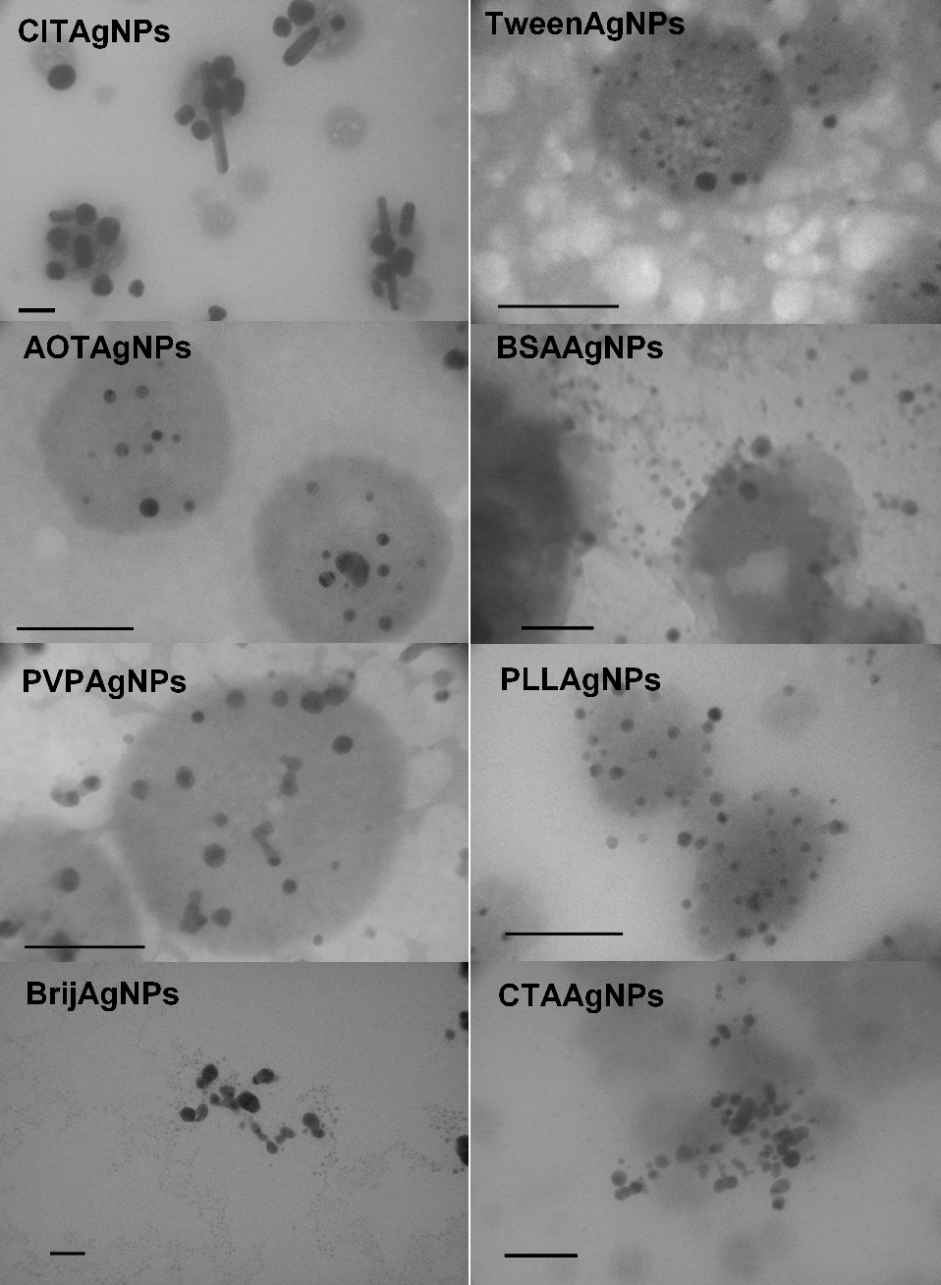
Transmission electron micrographs (TEM) of different silver nanoparticles coated with trisodium citrate (CITAgNP), sodium bis(2-ethylhexyl) sulfosuccinate (AOTAgNP), poly(vinylpyrrolidone) (PVPAgNP), Brij 35 (BrijAgNP), Tween 20 (TweenAgNP), bovine serum albumin (BSAAgNP), poly(L-lysine) (PLLAgNP)**,** and cetyltrimethylammonium bromide (CTAAgNP) in biological media supplemented with 0.1% bovine serum albumin (BMP) after 1 h. Scale bars are 100 nm.

A similar stabilizing effect of BSA against the aggregation of nanoparticles was previously reported [3,5,30,57–58]. Although the presence of BSA prevents NP agglomeration, the *d*_H_ obtained from size distributions by volume increased by a factor of two and more for all NPs upon suspension in BMP due to the bulky globular nature of the BSA coating. This can be seen by comparing data from Table 1, Table 2 and Table 3. The very heterogeneous size distribution for different NPs indicates non-uniform surface coverage depending on surface coating. Thus, the BSA molecules clustered and adsorbed on the NP surface variously for different AgNPs and SPIONs, resulting in a thickness variation. Moreover, the BSA coating is likely to include many surface regions that retain adsorbed coating agents, remaining from the original synthesis. For CITAgNPs, PVPAgNPs, TweenAgNPs, BSAAgNPs and PLLSPIONs, characterized by a bimodal size distribution in UW, interaction with BSA led to a monomodal size distribution in which all NPs were covered by several BSA molecules. For the NPs that were agglomerated in BM, the addition of BSA inhibited completely or significantly reduced the agglomeration process. It is clearly visible from Figure 5 that the *d*_H_ obtained from size distributions by volume was constant and significantly lower in BMP compared to BM for AOTAgNBPs, BrijAgNPs, PLLAgNPs, and CTAAgNPs after 1 h. The observed increase in the mean *d*_H_ for CITAgNPs, UNSPIONs, MANSPIONs and PLLSPIONs in the BMP after 1 h is not a result of agglomeration, but rather an indication of a slower adsorption of BSA molecules to NP surfaces depending on the interchange of the coating agent with BSA. Such an assumption was further confirmed by TEM that clearly showed non-agglomerated, well-dispersed NPs in the BMP (Figure 7). This highlights the difficulties of using the DLS technique for extracting changes in the actual size of NP core when taking into account surface coatings, which can agglomerate/cluster on the NP surface. BSA may be bound by a relatively strong covalent bond between the NPs surface and cysteine groups or via protein–protein electrostatic or depletion interactions. If both interactions take place simultaneously, the thickness of a PC will vary depending on the type of NP.

The ELS data showed that all of the NPs had very similar potential values in BMP regardless of the coating agent, ranging from −9.9 to −12.4 mV (Figure 4). The decrease of absolute values of the ζ-potential toward zero in the BMP compared to BM imply that the BSA coating itself was the main source of particle stability in the BMP, as this protein is just slightly negatively charged at physiological pH values. The measured ζ-potentials were very close to the values determined for pure BSA dispersions, −7.5 ± 0.04 mV, which is not surprising taking into account the relatively high protein concentration. Thus, BSA conjugates provided an enhanced electrostatic repulsion against the agglomeration of metallic NPs in DMEM. The BSA has negative charges above its isoelectric point (pH 4.78) [59] and the electrostatic forces dominate over hydrophobic interactions. Accordingly, the attractive forces between the positively charged AgNPs and the negatively charged BSA led to protein adsorption, but questions remained about why the repulsive forces between the negatively charged NPs and BSA did not prevent protein adsorption. Besides a negatively charged surface at physiological pH, the structure of BSA is also characterized by positively charged lysine and cysteine [60]. Therefore, its interaction with NPs is hardly trivial.

The most important observation of this study is that BSA enables a colloidal stabilization of metallic NPs in biological fluids regardless of their chemical composition, surface structure and surface charge. This is also evident from the micrographs typically visualized by TEM for NPs dispersed in BMP (Figure 7). These images show that NPs are well-dispersed, but can be found only on grid areas where drops of BMP settled. Only BSAAgNPs and BrijAgNPs were dispersed all over the TEM grid.

Our results are in good agreement with recently published data for stabilization of different metallic NPs in protein-containing media [3,54,61–64]. The mechanisms of PC adsorption and the way how the PC is arranged at the NP surface are crucial for gaining an understanding of the biological reactivity of NPs in vivo [61]. In principle, the protection against colloidal agglomeration offered by BSA could be used in different nanotechnological applications, but also highlights the facilitated transport of nanoparticles across the bloodstream. This study clearly shows that surface coating strongly affects colloidal stability and behavior of metallic NPs in biological environment as presented in Table 4.

**Table 4:** Summarized effects of different coating agents on the stability of silver and maghemite nanoparticles in model biological medium after 1 h. UW - ultrapure water, BM - biological cell culture medium without addition of protein, BMP - BM supplemented with common serum protein.

coating agent	BM compared with UW	BMP compared with BM

trisodium citrate (CIT)	stable dispersion, │ζ│ decreased, no morphology changes	stable dispersion, │ζ│ decreased, localized in BMP areas on TEM grid

sodium bis(2-ethylhexyl) sulfosuccinate (AOT)	pronounced aggregation, │ζ│ decreased, morphology changes (irregular shapes)	stable dispersion, │ζ│ decreased, localized in BMP areas

cetyltrimethylammonium bromide (CTA)	partial stabilization, │ζ│ decreased, charge reversal, morphology changes (irregular shapes)	stable dispersion, │ζ│ decreased, localized in BMP areas on TEM grid

Brij 35 (Brij)	pronounced aggregation, │ζ│ increased, morphology changes (irregular shapes)	stable dispersion, │ζ│ decreased, dispersed over the TEM grid

Tween 20 (Tween)	partial stabilization, │ζ│ increased, morphology changes (irregular shapes)	stable dispersion, │ζ│ decreased, localized in BMP areas on TEM grid

poly(vinylpyrrolidone) (PVP)	stable dispersion, │ζ│ decreased, no morphology changes	stable dispersion, │ζ│ decreased, localized in BMP areas on TEM grid

poly(L-lysine) (PLL)	pronounced aggregation for AgNPs, │ζ│ decreased, charge reversal, partial stabilization for SPIONs, │ζ│ increased, morphology changes (irregular shapes)	stable dispersion, │ζ│ increased, localized in BMP areas on TEM grid

bovine serum albumin (BSA)	stable dispersion, │ζ│ increased, no morphology changes	stable dispersion, │ζ│ decreased, dispersed over the TEM grid

D-mannose (MAN)	pronounced aggregation, │ζ│ decreased, morphology changes (irregular shapes)	stable dispersion │ζ│ decreased localized in BMP areas on TEM grid

### Behavior of NPs in blood and blood plasma

The implications of the PC on the bioactivity nanomaterials in vivo are enormous. Biological fluids are complex environments in which it is difficult even to predict all of the possible NP modifications and interactions. In such an environment, the dynamic adsorption of different biomolecules onto the surface of metallic NPs is a well-established fact, which irreversibly changes the nature of the original NPs [61,65].

**Figure 8:**
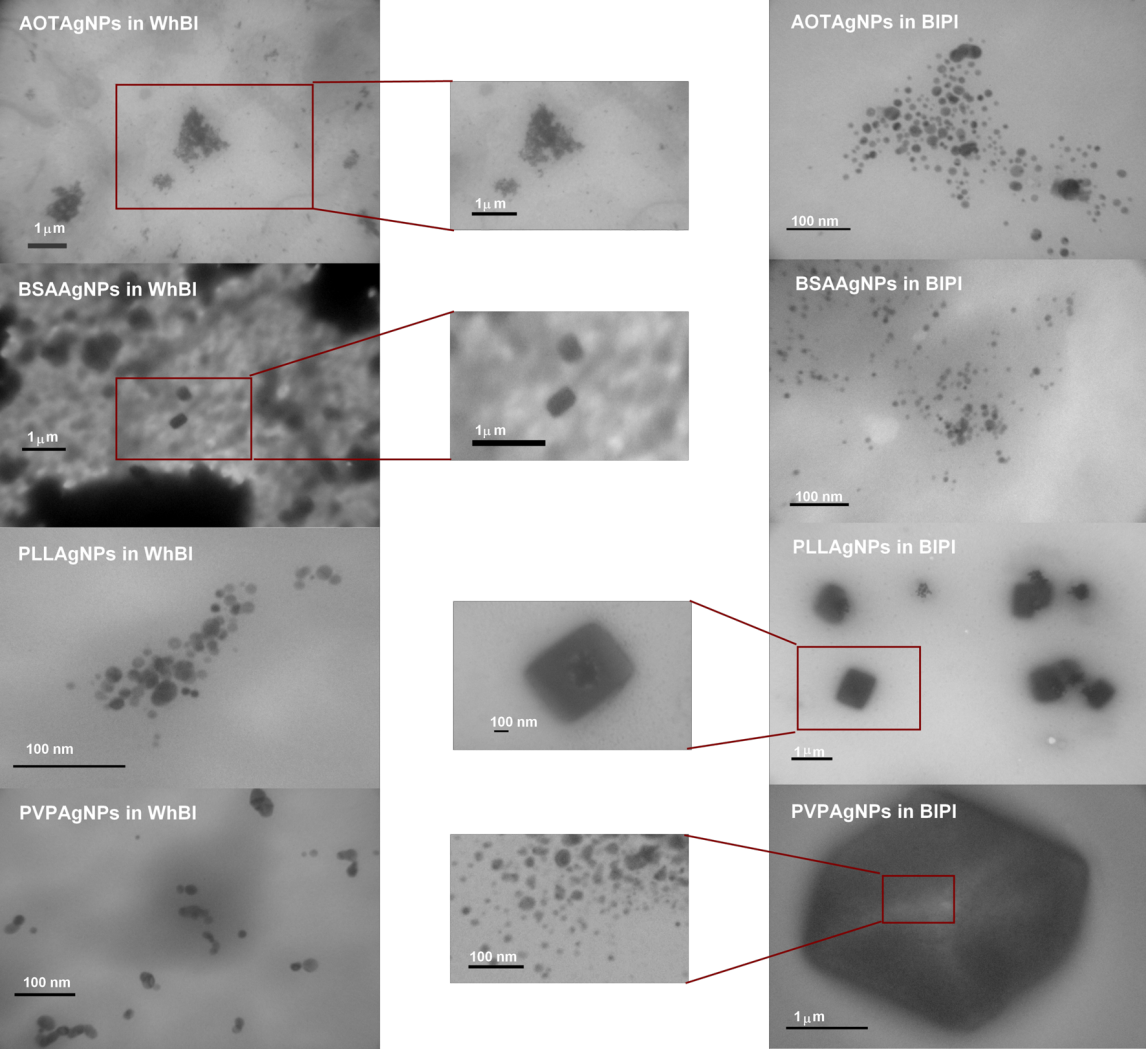
Transmission electron micrographs (TEM) of different silver nanoparticles coated with sodium bis(2-ethylhexyl) sulfosuccinate (AOTAgNP), bovine serum albumin (BSAAgNP), poly(L-lysine) (PLLAgNP), and poly(vinylpyrrolidone) (PVPAgNP) in whole blood (WhBl) and blood plasma (BlPl) of Wistar rats after 1 h.

In order to examine how differently coated metallic NPs behave in more complex biological fluids, PVPAgNPs, BSAAgNPs, AOTAgNPs and PLLAgNPs were dispersed in rat whole blood (WhBl) and blood plasma (BlPl). After incubation for 1 h, samples were examined by TEM as described in the Experimental section. The TEM micrographs showed rather unexpected features (Figure 8). All of the AgNPs except PVPAgNPs, which were initially small and exhibited spherical shape, were transformed depending on the media. AOTAgNPs and BSAAgNPs significantly changed their shape and size in WhBl, but stayed very well dispersed in BlPl. On the contrary, the morphology of PLLAgNPs and PVPAgNPs changed in BlPl, but remained unchanged in WhBl (Figure 8). The BSAAgNPs formed square- and rectangular-shaped agglomerates larger than 200 nm in WhBl. Similarly shaped structures were found for PLLAgNPs in BlPl, while PVPAgNPs formed large hexagonal nanocomposites in BlPl (Figure 8). Interestingly, AOTAgNPs were associated in triangle clusters formed of small, separated NPs (Figure 8).

It has been very well established that the shape of metallic NPs may be controlled using different surfactants [66–67]. The choice and addition of surfactants may successfully control the synthesis of nanodiscs, triangular nanoplates or nanospheres. In recent years, solution-phase methods developed rapidly toward a reproducible preparation of metallic NPs with controlled shape [66]. A typical synthesis of nanocrystals can be divided into three levels: nucleation, evolution of nuclei into seed, and growth of seed into nanocrystals. The mechanism behind such a synthesis is extremely complicated, but the type of coating agent proved to be crucial for the final shape of a nanocrystal [66]. The micrographs presented in Figure 8 suggest that our initially small AgNPs appeared as seeds in WhBl or BlPl, where further growth to nanocomposites was accomplished. Thus, our results indicate an in vivo synthesis of metallic nanocrystals in mammalian organisms, similarly to that already described in microorganisms [68].

There are many examples of in vivo formation of nanomaterials (NMs) in biological systems [68]. The most common process is the biomineralization of bones and shells [69]. It is interesting that the shape of these bionanomaterials is usually induced by an engulfing organic matrix [69]. Different microorganisms, such as magnetotactic bacteria or diatoms, are also able to produce nanocrystals in vivo [70–74]. The biosynthesis of metallic NMs with controlled morphology is governed by using different bacterial strains [68]. For example, *Pseudomonas stutzeri* AG259, a metal-accumulating bacterium, is able to synthesize AgNPs through its detoxification process after exposure to silver [75]. It is somewhat intriguing that we observed similar AgNPs forms in WhBl and BlPl (Figure 8) as already described for *Pseudomonas stutzeri* AG259 [75].

The reason for the observed differences between WhBl and BlPl is unclear, but it implies that NP stability and morphology can be significantly changed with only small changes in the composition of the biological medium. Our results point out that an accurate characterization of physicochemical parameters and behavior of NPs in a particular biological environment is imperative for clinical relevance to target organ groups. As a consequence, the development of nanomaterials for theragnostics is an ambitious goal with many parameters to assess.

## Conclusion

The lack of fundamental knowledge about the biocompatibility of metal-based nanomaterials and their effect and behavior in biological systems may restrict the capability to establish principles used as regulatory guidance and design safe nanomaterials. The detection and assessment of the colloidal stability of metallic NPs is vital. The presented work describes a systematically conducted experimental approach consisting of techniques that, although simple, are sufficient to perform a fast screening of the biocompatibility and colloidal stability of metallic NPs in biological environments. The obtained results have shown that the agglomeration behavior of metallic NPs in aqueous solutions with the pH and ionic strength close to biological fluids depends on the surface coating. This study confirmed that the presence of proteins such as BSA plays a major role in the colloidal stabilization of metallic NPs in biological fluids. Data on the behavior of differently coated NPs in whole blood and blood plasma highlights the importance of investigating the behavior and effects of metallic NPs in a variety of biological fluids in addition to including as many of the NPs properties as possible. It is not superfluous to stress that a systematic study of the stability and behavior for various NPs in addition to the best possible characterization of NPs would enable clear conclusions and predictions about the effects of NPs in a variety of biological systems.

## Experimental

### Chemicals and materials

If not otherwise stated, chemicals were obtained from Sigma-Aldrich Chemie GmbH (Munich, Germany). Dulbecco’s modified Eagle’s medium (DMEM) with 4.5 g·L^−1^ glucose without L-glutamine and sodium dihydrogen phosphate as buffering agent (product number 12-614Q) was obtained from Lonza (Verviers, Belgium). Bovine serum albumin (product number A-7906, Sigma-Aldrich Chemie GmbH, Steinheim, Germany) was used as received without further purification. The plastic and glassware used for chemical analysis were from Sarstedt (Belgium). Osmium tetroxide was purchased from Agar Scientific (Stansted, UK) and TAAB epoxy resin (medium hard) from Aldermaston (Berkshire, UK). All dilutions were made with ultrapure water (18.2 MΩ·cm), obtained from a GenPureUltraPure water system (TKA Wasseraufbereitungssysteme GmbH, Niederelbert, Germany).

### Synthesis of metallic nanoparticles

The syntheses of AgNPs and SPIONs with different surface coatings were conducted as previously described [49] using structurally diverse surface coatings: trisodium citrate (CIT), sodium bis(2-ethylhexyl) sulfosuccinate (AOT), cetyltrimethylammonium bromide (CTA), poly(vinylpyrrolidone) (PVP), poly(L-lysine) (PLL), bovine serum albumin (BSA), Brij 35 (Brij), Tween 20 (Tween) and D-mannose (MAN).

Silver nanoparticles coated with sodium bis(2-ethylhexyl) sulfosuccinate (AOTAgNP), cetyltrimethylammonium bromide (CTAAgNP), poly(vinylpyrrolidone) (PVPAgNP), poly(L-lysine)(PLLAgNP), and Tween 20 (TweenAgNP) were synthesized by reducing AgNO_3_ with NaBH_4_. Briefly, the solutions of capping agent were prepared by dissolving appropriate amounts of capping agent in ultra-pure water. Then, 9.2 mL of 50 mM AgNO_3_ was added dropwise and dissolved by constant stirring on an IKA RCT basic magnetic stirrer plate (IKA Werke, Germany). To this solution, a volume of 2 mL of 0.4 M NaBH_4_ solution was added dropwise (about 1 drop/s). The final concentrations of AOT, CTAB, PVP, PLL, and Tween were 500, 500, 75, 20 and 6 mM, respectively. The reaction mixture was stirred vigorously at room temperature for 45 min. After the synthesis, silver colloids were centrifuged at 11,000*g* for 20 min. After decanting the supernatant, the residue was suspended in ultrapure water and kept at 4 °C in the dark. BrijAgNPs were synthesized by mixing an aqueous solution of AgNO_3_ (0.09 mL, 50 mM), Brij 35 (5 mL, 0.45 mM) and hydrogen peroxide (0.105 mL, 30 wt %) with 44.5 mL ultrapure water. The mixture was vigorously stirred at room temperature in the presence of air. The final volume was kept at 50 mL. To this mixture, NaBH_4_ (0.4 mL, 200 mM) was rapidly injected, generating a colloid that was pale yellow. After 30 min, the colloid darkened to a deep-yellow color indicating the formation of AgNPs. CITAgNPs were synthesized via the following protocol: 200 μL of the aqueous solution of ascorbic acid (AsA) with a concentration of 0.1 mM was added into 190 mL of boiling water, followed by boiling for an additional 1 min. Then, 3.8 mL of the aqueous solution of sodium citrate (35.4 mM) and 1.2 mL of the aqueous solution of AgNO_3_ (50 mM) were consecutively added to 5 mL of water under stirring at room temperature. After 5 min of incubation at room temperature, the citrate–AgNP mixture solution was injected into the boiling aqueous solutions of AsA (just after 1 min boiling after AsA addition to boiling water). The final concentrations of reactants were 0.673 mM for sodium citrate, 0.3 mM for AgNO_3_ and 0.1 μM for AsA. The color of the reaction solution quickly changed from colorless to yellow. The transparent and yellow reaction solution was further boiled for 1 h under stirring to warrant formation of uniform quasi-spherical AgNPs. Purification of AgNPs was performed by centrifugation of colloidal solution two times at 11,000*g* for 30 min. Supernatant was decanted and precipitate was redispersed in ultrapure water by sonification. Silver nanoparticles directly conjugated to bovine serum albumin (BSAAgNPs) were prepared as follows: 7.6 mL of 50 mM AgNO_3_ was added dropwise under stirring to 33 mL of ultrapure water containing dissolved 90 mg of BSA. Then, sodium borohydride (1 mL, 0.397 M) was added to an aqueous solution of AgNO_3_ and BSA under vigorous stirring. The molar ratio of Ag^+^:BSA and 
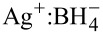
 were 28:1 and 1:1, respectively. The reaction volume was 40 mL, and contained 13.50 μmol BSA. The reaction was allowed to proceed for 1 h, and the product was purified by precipitation at −5 °C using ultracentrifugation.

Three different maghemite nanoparticles (γ-Fe_2_O_3_NPs), uncoated, coated with poly(L-lysine) and D-mannose, were prepared by coprecipitation of FeCl_2_ and FeCl_3_ using ammonium hydroxide, followed by the oxidation of the resulting magnetite with sodium hypochlorite [46–47]. The obtained superparamagnetic maghemite (γ-Fe_2_0_3_) was referred as uncoated γ-Fe_2_O_3_NPs (UNSPIONs). The post-synthesis coating of maghemite with D-mannose (MANSPIONs) or poly(L-lysine) (PLLSPIONs) was achieved [46] by addition of D-mannose or poly(L-lysine) to the primary uncoated maghemite cores [4].

### Analytical methods

As described in [49], the size and charge of NPs were measured by dynamic (DLS) and electrophoretic light scattering (ELS), respectively, using Zetasizer Nano ZS (Malvern, UK). Visualization of NPs were done using a transmission electron microscope (TEM, Zeiss 902A). Total silver concentrations in AgNPs were determined using an Agilent Technologies 7500cx inductively coupled plasma mass spectrometer (ICPMS) (Waldbronn, Germany).

### Characterization of nanoparticles and dispersion protocols

Careful characterization and colloidal stability evaluation of each NP type was conducted using several different dispersion protocols: ultrapure water (UW), DMEM high glucose as model biological medium (BM), BM supplemented with 0.1 or 1% BSA (BMP), whole blood (WhBl) and blood plasma (BlPl) taken from the Wistar rat. The aim was to investigate the behavior of each NP after 1 h in different biological environments. In each dispersion experiment, NPs were applied at total metal concentration of 1 or 10 mg·L^−1^.

The stock solution of BSA in DMEM was freshly prepared for each experiment and then diluted to the desired concentrations. Differently coated metallic NPs were dispersed in BSA solutions to the final concentration of 1 mg·L^−1^ just before DLS measurements. Among the most important parameters of colloidal systems is their particle size, which can be used as an indicator of their stability. DLS is the most common and versatile technique for measuring size distribution of NPs in solutions (Murdock et al. [53]). However, conventional DLS has its limitations. The main interferences in the biological matrix originate from the light scattering of different biological components and a mixture of different sizes of fractal-shaped agglomerates. In our model BMP system, the effect of BSA scattering requires cautious and thoughtful analysis of DLS results. The pure BSA in DMEM had a volume-weighted mean size of 7.4 ± 0.8 nm, while *d*_H_ obtained from size distributions by intensity was shown to be 9.6 ± 0.9 nm, as expected for a globular protein of 66 kDa [60]. Thus, the BSA scattering is the most significant in interpretation of DLS results for small, non-agglomerated NPs with sizes close to BSA. That was not the issue in the present study. The size of the measured metallic NPs in all BMP systems was at least two-fold larger than *d*_H_ of BSA therefore no overlaps of the peak maximums were observed. On the other hand, due to the low concentration of metallic NP, the volume peak area (%) in all of the BMP systems was significantly smaller compared to BSA. Conversely, size distribution by intensity showed more realistic peak ratios. To address this problem to some extent, BSA levels 2.4 (1% BSA) and 24 (0.1% BSA) times lower than physiological concentrations ([BSA] = 375 μM) were added to the BM (in order to prepare BMPs). In order to present results in a transparent way and obtain accurate conclusions from the DLS measurements, size distribution by intensity and volume were used in analyzing the results. Intensity-weighted size distribution is the first order result from a DLS experiment calculated from the scatter intensity of each particle in solution. On the other hand, intensity distributions can be biased towards larger particles since the intensity of particle light scatter varies with the 6th power of particle diameter. In order to avoid overestimations arising from the scattering of larger particles, volume-weighted size distributions are often used. In addition, *d*_H_ obtained from size distributions by volume was presented so results could be comparable with our previously published studies. It should be noted that the data was sometimes compiled from different synthesis batches of NPs, leading to some discrepancy in the size distributions of the various control samples.

For dispersions of NPs in whole blood (WhBl) and blood plasma (BlPl), whole blood and blood plasma were obtained from healthy twelve weeks old male Wistar rats. Animals were killed by narketan/xilapan anesthesia following the whole blood collection by cardiac puncture. The experiment was approved by the Ethics Committee for Animal Studies of the Institute for Medical Research and Occupational Health according to European and Croatian legislation on animal experimentation and International Council for Laboratory Animal Science ethical guidelines for researchers, respectively. Then, different NPs were dispersed in 1 mL of WhBl or BlPl at a final metal concentration of 10 mg·L^−1^ and agitated for 1 h on a digital waving rotator (Thermo Scientific, USA). After incubation, suspensions were diluted 50 times before further analysis. It should be noted that DLS and ELS measurements were impossible in WhBl and BlPl suspensions.

TEM samples were prepared by depositing a drop of the NPs suspension after 1 h of incubation at room temperature on a Formvar^®^ coated copper grid and air-drying at room temperature.

## References

[1] Tai J-T, Lai C-S, Ho H-C, Yeh Y-S, Wang H-F, Ho R-M, Tsai D-H (2014). Langmuir.

[2] Lohse S E, Murphy C J (2012). J Am Chem Soc.

[3] Dominguez-Medina S, Blankenburg J, Olson J, Landes C F, Link S (2013). ACS Sustainable Chem Eng.

[4] Babič M, Horák D, Trchová M, Jendelová P, Glogarová K, Lesný P, Herynek V, Hájek M, Syková E (2008). Bioconjugate Chem.

[5] Kittler S, Greulich C, Gebauer J S, Diendorf J, Treuel L, Ruiz L, Gonzalez-Calbet J M, Vallet-Regi M, Zellner R, Köller M (2010). J Mater Chem.

[6] Yen H-J, Hsu S-H, Tsai C-L (2009). Small.

[7] Liu J Y, Hurt R H (2010). Environ Sci Technol.

[8] Walters C, Pool E, Somerset V (2013). Toxicol Environ Chem.

[9] Pettibone J M, Gigault J, Hackley V A (2013). ACS Nano.

[10] MacCuspie R I, Allen A J, Hackley V A (2011). Nanotoxicology.

[11] Loza K, Diendorf J, Sengstock C, Ruiz-Gonzalez L, Gonzalez-Calbet J M, Vallet-Regi M, Köller M, Epple M (2014). J Mater Chem B.

[12] Liu J Y, Sonshine D A, Shervani S, Hurt R H (2010). ACS Nano.

[13] Liu J Y, Wang Z Y, Liu F D, Kane A B, Hurt R H (2012). ACS Nano.

[14] Stebounova L V, Guio E, Grassian V H (2011). J Nanopart Res.

[15] Hotze E M, Labille J, Alvarez P, Wiesner M R (2008). Environ Sci Technol.

[16] Hussain S M, Braydich-Stolle L K, Schrand A M, Murdock R C, Yu K O, Mattie D M, Schlager J J, Terrones M (2009). Adv Mater.

[17] Park E-J, Yi J, Kim Y, Choi K, Park K (2010). Toxicol In Vitro.

[18] Zook J M, MacCuspie R I, Locascio L E, Halter M D, Elliott J T (2011). Nanotoxicology.

[19] Tejamaya M, Römer I, Merrifield R C, Lead J R (2012). Environ Sci Technol.

[20] Vidic J, Haque F, Guigner J M, Vidy A, Chevalier C, Stankic S (2014). Langmuir.

[21] Marucco A, Catalano F, Fenoglio I, Turci F, Martra G, Fubini B (2015). Chem Res Toxicol.

[22] Leo B F, Chen S, Kyo Y, Herpoldt K-L, Terrill N J, Dunlop I E, McPhail D S, Shaffer M S, Schwander S, Gow A (2013). Environ Sci Technol.

[23] Li X, Lenhart J J, Walker H W (2012). Langmuir.

[24] MacCuspie R I (2011). J Nanopart Res.

[25] Sharma V K, Siskova K M, Zboril R, Gardea-Torresdey J L (2014). Adv Colloid Interface Sci.

[26] Jiang J, Oberdörster G, Biswas P (2009). J Nanopart Res.

[27] Gebauer J S, Treuel L (2011). J Colloid Interface Sci.

[28] Thanh N T K, Rosenzweig Z (2002). Anal Chem.

[29] Schulze C, Kroll A, Lehr C-M, Schäfer U F, Becker K, Schnekenburger J, Schulze Isfort C, Landsiedel R, Wohlleben W (2008). Nanotoxicology.

[30] Gebauer J S, Malissek M, Simon S, Knauer S K, Maskos M, Stauber R H, Peukert W, Treuel L (2012). Langmuir.

[31] Segets D, Marczak R, Schäfer S, Paula C, Gnichwitz J-F, Hirsch A, Peukert W (2011). ACS Nano.

[32] Kohut A, Voronov A, Peukert W (2007). Langmuir.

[33] Gilbert B, Huang F, Zhang H, Waychunas G A, Banfield J F (2004). Science.

[34] Min Y, Akbulut M, Kristiansen K, Golan Y, Israelachvili J (2008). Nat Mater.

[35] Zook J M, Halter M D, Cleveland D, Long S E (2012). J Nanopart Res.

[36] Treuel L, Nienhaus G U (2012). Biophys Rev.

[37] Walczyk D, Bombelli F B, Monopoli M P, Lynch I, Dawson K A (2010). J Am Chem Soc.

[38] Moerz S T, Huber P (2014). Langmuir.

[39] Monopoli M P, Walczyk D, Campbell A, Elia G, Lynch I, Bombelli F B, Dawson K A (2011). J Am Chem Soc.

[40] Lynch I, Salvati A, Dawson K A (2009). Nat Nanotechnol.

[41] Shannahan J H, Lai X, Ke P C, Podila R, Brown J M, Witzmann F A (2013). PLoS One.

[42] Park M V D Z, Neigh A M, Vermeulen J P, de la Fonteyne L J J, Verharen H W, Briedé J J, van Loveren H, de Jong W H (2011). Biomaterials.

[43] El Badawy A M, Silva R G, Morris B, Scheckel K G, Suidan M T, Tolaymat T M (2011). Environ Sci Technol.

[R44] 44European Commission. Communication from the Commission to the European Parliament, the Council and the European Economic and Social Committee. Second regulatory review on nanomaterials. Brussels, 3.10.2012, COM (2012) 572 final.

[45] Martin M N, Allen A J, MacCuspie R I, Hackley V A (2014). Langmuir.

[46] Horák D, Babič M, Jendelová P, Herynek V, Trchová M, Pientka Z, Pollert E, Hájek M, Syková E (2007). Bioconjugate Chem.

[47] Horák D, Babič M, Jendelová P, Herynek V, Trchová M, Likavčanová K, Kapcalová M, Hájek M, Syková E (2009). J Magn Magn Mater.

[48] Vinković Vrček I, Žuntar I, Petlevski R, Pavičić I, Dutour Sikirić M, Ćurlin M, Goessler W (2014). Environ Toxicol.

[49] Vinković Vrček I, Pavičić I, Crnković T, Jurašin D, Babič M, Horák D, Lovrić M, Ferhatović L, Ćurlin M, Gajović S (2015). RSC Adv.

[50] Milić M, Leitinger G, Pavičić I, Zebić Avdičević M, Dobrović S, Goessler W, Vinković Vrček I (2015). J Appl Toxicol.

[51] Kvítek L, Panáček A, Soukupová J, Kolář M, Večeřová R, Prucek R, Holecová M, Zbořil R (2008). J Phys Chem C.

[52] Churchman A H, Wallace R, Milne S J, Brown A P, Brydson R, Beales P A (2013). Chem Commun.

[53] Murdock R C, Braydich-Stolle L, Schrand A M, Schlager J J, Hussain S M (2008). Toxicol Sci.

[54] Cedervall T, Lynch I, Lindman S, Berggård T, Thulin E, Nilsson H, Dawson K A, Linse S (2007). Proc Natl Acad Sci U S A.

[55] Maiorano G, Sabella S, Sorce B, Brunetti V, Malvindi M A, Cingolani R, Pompa P P (2010). ACS Nano.

[56] Simón-Vázquez R, Lozano-Fernández T, Peleteiro-Olmedo M, González-Fernández Á (2014). Colloids Surf, B.

[57] Ravindran A, Singh A, Raichur A M, Chandrasekaran N, Mukherjee A (2010). Colloids Surf, B.

[58] Yang Q, Liang J, Han H (2009). J Phys Chem B.

[59] Patil S, Sandberg A, Heckert E, Self W, Seal S (2007). Biomaterials.

[60] Peters T (1996). All About Albumin: Biochemistry, Genetics and Medical Applications.

[61] Saptarshi S R, Duschl A, Lopata A L (2013). J Nanobiotechnol.

[62] Dobrovolskaia M A, Patri A K, Zheng J, Clogston J, Ayub D, Aggarwal P, Neun B W, Hall J B, McNeil S E (2009). Nanomedicine.

[63] Alkilany A M, Nagaria P K, Hexel C R, Shaw T J, Murphy C J, Wyatt M D (2009). Small.

[64] Khullar P, Singh V, Mahal A, Dave P N, Thakur S, Kaur G, Singh J, Singh Kamboj S, Singh Bakshi M (2012). J Phys Chem C.

[65] Casals E, Pfaller T, Duschl A, Oostingh G J, Puntes V (2010). ACS Nano.

[66] Xia Y, Xiong Y, Lim B, Skrabalak S E (2008). Angew Chem, Int Ed.

[67] Goesmann H, Feldmann C (2010). Angew Chem, Int Ed.

[68] Klaus-Joerger T, Joerger R, Olsson E, Granqvist C-G (2001). Trends Biotechnol.

[69] Lowenstam H A (1981). Science.

[70] Spring S, Schleifer K-H (1995). Syst Appl Microbiol.

[71] Schüler D, Frankel R B (1999). Appl Microbiol Biotechnol.

[72] Kajander E O, Çiftçioglu N (1998). Proc Natl Acad Sci U S A.

[73] Rivadeneyra M-A, Delgado G, Soriano M, Ramos-Cormenzana A, Delgado R (1999). Curr Microbiol.

[74] Keefe W E (1976). Infect Immun.

[75] Klaus T, Joerger R, Olsson E, Granqvist C-G (1999). Proc Natl Acad Sci U S A.

